# The Combination of MIF Inhibitor and AEP Targeted Inhibitor to Reduce Lung Metastasis in Breast Cancer and Its Mechanism

**DOI:** 10.1111/jcmm.70616

**Published:** 2025-05-24

**Authors:** Junsong Chen, Wenke Xu, Luyang Meng, Xin Zhang, Mengyao Lin, Sheng Zhang, Yi Liu, Fang Guo

**Affiliations:** ^1^ Key Laboratory of Systems Biomedicine (Ministry of Education), Shanghai Center for Systems Biomedicine, Shanghai Jiao Tong University Shanghai China; ^2^ Department of Vascular Surgery Hangzhou Third People's Hospital Hangzhou Zhejiang China; ^3^ Department of Pathology The First Affiliated Hospital of Fujian Medical University Fuzhou China; ^4^ Department of Radiotherapy Huangpu Branch of the Ninth People's Hospital, Shanghai Jiaotong University School of Medicine Shanghai China

**Keywords:** asparagine endopeptidase, breast cancer, CD74, lung metastasis, macrophage migration inhibitory factor

## Abstract

Breast cancer, the most prevalent malignant tumour in women, is characterised by high metastatic potential and frequent recurrence, both of which significantly impact patient prognosis following metastasis. To address this challenge, identifying novel therapeutic target combinations is critical for improving metastatic breast cancer treatment. This study investigates the mechanism by which asparaginyl endopeptidase (AEP) regulates breast cancer metastasis. Bioinformatics analysis revealed a potential interaction between AEP and CD74, which was subsequently confirmed through co‐immunoprecipitation (co‐IP) experiments. Further investigations demonstrated that AEP activates ERK pathway phosphorylation via CD74 regulation, thereby enhancing epithelial‐mesenchymal transition (EMT) progression and promoting breast cancer cell migration. Compared to controls, dual inhibition of AEP and CD74 effectively reduced EMT markers and the migratory capacity of cancer cells in vitro. Subsequent in vivo experiments showed that this combinatorial strategy significantly suppressed breast cancer lung metastasis in mice without observable toxicity. These findings elucidate the molecular mechanism through which AEP promotes metastasis via CD74 regulation, while validating the therapeutic efficacy and safety of dual AEP/CD74 targeting. This study provides a novel conceptual framework and potential therapeutic targets for metastatic breast cancer intervention.

## 1 Introduction

Breast cancer has become the leading cause of death and health concerns for women worldwide. It is characterised by high metastasis and poor prognosis. When breast cancer metastasises, the overall survival period is reduced to 2–3 years, with a 5‐year survival rate of only 27% [1]. This greatly impacts patient prognosis. The most common distal metastasis sites of breast cancer primarily involve bone, lung, brain, and liver. Approximately 21%–36% of breast cancer patients experience lung metastasis, with 36.9% of them having triple‐negative breast cancer [2]. The metastasis process in breast cancer typically involves several steps. First, tumour cells undergo epithelial mesenchymal transition (EMT), acquiring a mesenchymal phenotype [3]. This enhances their ability to migrate, invade, and degrade the extracellular matrix [4]. Subsequently, they detach from the primary tumour, infiltrate the surrounding tissue, and penetrate the tissue base to invade blood vessels or lymphatic vessels. Through the circulatory system, they spread to distant organs, interact with cells in those organs, and form micro metastatic foci, ultimately colonising the distant organs [5]. During this process, breast cancer cell EMT progression is modulated by key signalling pathways including AKT, ERK, and STAT3. These pathways converge to regulate transcription factors such as Twist1, SLUG, Snail, ZEB1, and ZEB2, ultimately governing the expression and transcriptional activity of EMT‐associated proteins [3]. Cysteine cathepsins comprise a family of proteases that have been widely implicated in the invasion and progression of primary breast tumours. Legumain (LGMN), also known as asparagine endopeptidase (AEP), is a lysosomal cysteine endoprotease originally identified in the seeds of legumes [6]. It is the only mammalian enzyme that cleaves C‐terminally to asparagine residues [7]. AEP is overexpressed on the cell surface and in cytoplasmic vesicles of solid tumours and is associated with the development, invasion, and metastasis stages in several cancers [8], including breast [9], prostate, colorectal [10], and gastric carcinomas. Mechanistically, AEP exerts pro‐metastatic effects in tumour cells primarily through its interaction with AKT and ERK pathways—critical pathways driving tumour progression. Furthermore, AEP facilitates metastasis by dual mechanisms: mediating extracellular matrix (ECM) degradation and remodelling the tumour microenvironment (TME) [6, 8]. Numerous studies have confirmed that AEP is a potential prognostic factor in breast cancer and that its expression significantly correlates with a shortened lifespan in patients, suggesting that AEP may play an important role in breast cancer invasion and metastasis [8]. We have identified a novel AEP inhibitor that effectively inhibits legumain enzyme activity in both in vitro and in vivo studies [11]. Additionally, targeted AEP inhibitors combined with chemotherapy drugs can significantly reduce the occurrence of bone metastasis in breast cancer and prolong the survival period of mice, by regulating EMT‐related genes and osteoclasts. Previous research has achieved promising results in the treatment of bone metastasis in breast cancer through the regulation of AEP expression [12]. However, studies have also found that epirubicin, when used as a combination chemotherapy drug, has certain toxic effects in mouse models, including weight loss and poor survival status. Therefore, finding a new target inhibitor to replace chemotherapy drugs has become a new approach to prevent and treat breast cancer metastasis.

Research on the MHC‐II complex revealed that AEP proteolytically processes the invariant chain CD74 during antigen presentation, thereby impairing MHC‐II‐mediated antigen presentation [13]. This finding definitively established CD74 as a canonical substrate of AEP. Clinically, elevated CD74 expression in malignancies exhibits a strong correlation with adverse clinical outcomes, driving growing research interest in its oncogenic functions across tumorigenesis and progression. Macrophage migration inhibitory factor (MIF) is a well‐defined, multi‐potent pro‐inflammatory mediator that plays an important role in both the innate and adaptive immune systems [14]. As an important receptor of MIF, CD74 initiates a cascade reaction of continuous ERK1/2 MAPK activation upon MIF activation [15]. MIF is associated with cancer occurrence and serves as a link between chronic inflammation and cancer [16]. MIF levels are significantly elevated in many tumours and promote cell proliferation in prostate tumours, breast cancer, melanoma, colon cancer, and glioma [17, 18]. Some studies have found that MIF from breast cancer patients' breast tissue has an anti‐inflammatory effect and can play a cancer‐promoting role in the interaction between cancer cells and the surrounding tissue, leading to a poor prognosis for patients [19]. The role of MIF/CD74 in lung metastasis of breast cancer and whether the combination of MIF inhibitors and AEP inhibitors is safer and more effective than the chemotherapy drug combination regimen used in previous studies (AEP inhibitors + epirubicin) are still unknown.

## 2 Materials and Methods

### 2.1 Cell Lines and Mice

HEK293T (RRID:CVCL_0063), mice breast cancer cell line (4 T1.2 (ATCC; CRL‐3406), 4 T1 (ATCC; CRL‐2539)) and human breast cancer cell line (MDA‐MB‐231 (ATCC; CRM‐HTB‐26), MCF‐7 (ATCC; HTB‐22)) were obtained from the American Type Culture Collection (ATCC, Manassas, VA). These cell lines were detected by STR (Short Tandem Repeat) in the past three years, and comparing the results with professional STR databases, the true identity of the tested cells can be authenticated. All cell lines were cultured in DMEM (Invitrogen), supplemented with 10% fetal bovine serum (FBS, Gibco), streptomycin (100 mg/mL), and penicillin (100 U/mL). The cells were cultured at 37°C with 5% CO_2_. 6–8 weeks old female BALB/c mice were bought from the Laboratory Animal Center of Shanghai Jiao Tong University, and all experiments were performed with mycoplasma‐free cells. All mice experiments were implemented under specific pathogen‐free (SPF) conditions and approved by Shanghai Jiao Tong University Animal Ethics Committee (approval number: 202201078).

### 2.2 Generation of Knockdown Cells

Stable cell lines with knockdown or overexpression of AEP were generated after lentiviral infection. The Lenti‐CRISPRV2‐Legumain recombinant plasmid was constructed and verified by DNA sequencing. The HEK293T cells were transfected with packaging vectors the lentiviruses were packaged using Lent‐iCRISPRV2‐Legumain, psPAX2 and pMD2.G, a three‐plasmid system using Lipofectamine 2000 (Invitrogen) according to the manufacturer's instructions. Lenti‐CRISPRV2‐Scramble serves as control, After 48 h, the supernatant containing virus was collected and used for infection of breast cancer cell lines. As a control, the Lenti‐CRISPRV2‐Scramble vector was used. Infection was done for 24 h in the presence of 8 μg/mL Polybrene. To obtain stable cell lines, cells were recovered with fresh medium and selected for 48 h with 4 μg/mL puromycin.

### 2.3 Cell Viability, Wound Healing Assays and Invasion Assay

A count of 1 × 10^3^ cells/well was plated into 96‐well plates in triplicate and was allowed to adhere overnight. After adherence, the medium was replaced with fresh medium containing BIC‐113 (1 μM), ISO‐1 (10 μM), BIC‐113 (1 μM) + ISO‐1 (10 μM) respectively. The cells were then incubated at 37°C for the indicated times. Then 10 μL/100 μL of CCK8 solution was added to each well to co‐incubate for 30 min at 37°C, and cell viability was measured spectrophotometrically at 450 nm. Wound healing assays: A confluent monolayer of cells was cultured overnight and a scratch was introduced with a pipette tip, and images of cell migration into the wound were captured at 0 and 24 h using a light microscope. The results are expressed as follows: the percentage of wound healing (%). Invasion assay: A density of 5 × 10^4^ cells/well was placed into the upper chamber of Transwell containing serum‐free medium with different concentrations of drugs of cells were allowed to migrate for 24 h, challenged by 10% FBS in the lower well. Following incubation, migrated cells on the lower side were fixed and stained using 0.5% crystal violet and quantified by counting cells from 5 captured images per well.

### 2.4 In Vivo Lung Metastasis Assay

4 T1 tumour cells (5 × 10^4^/100 μL) were injected into the caudal vein of female BALB/c mice using a 27‐gauge needle as described. On day 3, mice were randomly divided into 4 groups, treated with the dose of 5 mg/kg ISO‐1 alone, or 10 mg/kg BIC‐113 alone, combined 5 mg/kg ISO‐1 and 10 mg/kg BIC‐113 respectively with normal saline being given to the control group (seven mice per group). The mice were treated once every 3 days and weighed. After 15 days of treatment when the mice first showed signs of distress, the five viscera of the heart, liver, spleen, lung, and kidney were taken out from each group of mice and weighed. Then tissue samples were fixed in 10% neutral‐buffered formalin, decalcified, embedded in paraffin, and subjected to staining with haematoxylin and eosin (H&E, Sigma) or performed IHC testing. The expression of E‐cadherin, AEP, and CD74 was examined in the lung metastases of each group of mice. Antibodies: anti‐E‐cadherin, anti‐AEP, and anti‐CD74 (Santa cruz).

### 2.5 Protein Isolation and Western Blotting

Cell lysates from cultured cells or tumour cells were prepared with RIPA lysis buffer in the presence of protease inhibitor cocktails as described previously. To detect the expression of proteins, equivalent amounts of total protein were loaded for western blotting to analyse the target protein levels with indicated antibodies after BCA quantification of protein. Proteins were detected using an ECL chemiluminescence kit (Millipore, Billerica, MA). Densitometry analyses were carried out to determine band intensity using ImageJ software. Antibodies: anti‐E‐cadherin, anti‐N‐cadherin, anti‐p‐Akt, anti‐p‐p44/42, anti‐anti‐p44/42, and p‐NF‐κB (Cell Signal Technology), anti‐MMP2, anti‐AEP, and anti‐CD74 (abclonal), anti‐Akt, anti‐NF‐κB, HRP‐anti‐GAPDH, and HRP‐anti‐α‐Tubulin (60004, ProteinTech).

### 2.6 Co‐Immunoprecipitation and Cell Co‐Culture

After cell lysis and BCA quantification, add antibody to cell lysates, then bind overnight at 4°C. Proteins were loaded for western blotting to analyse the target protein levels with indicated antibodies after adding beads for 6 h. Antibodies: anti‐AEP, anti‐CD74 (Santa cruz). A density of 10^5^ cells/well was placed into the 6 well plates, then a density of 5 × 10^4^ cells/well was placed into the upper chamber of the transwell; both of them were cultured in 10% FBS DMEM for 48 h.

### 2.7 Elisa

Centrifuged (1000 rpm, 4°C, 5 min) the medium of 293 T cells after 24 h of culture and the supernatants were extracted. Then assayed for AEP expression in supernatants by Legumain (total) ELISA Kit EK1566 (BOSTER).

### 2.8 Statistical Analysis

All experimental data are expressed as mean standard error of mean ± (SEM). Statistical significance was determined by analysis of variance test. Statistical evaluation was carried out by Student's *t*‐test or one‐way ANOVA. Significance of difference was indicated as *p < 0.05 and **p < 0.01. The statistical analysis was performed by program Prism (GraphPad Software).

## 3 Results

### 3.1 AEP Regulates CD74 Expression by Interacting With CD74

**FIGURE 1:**
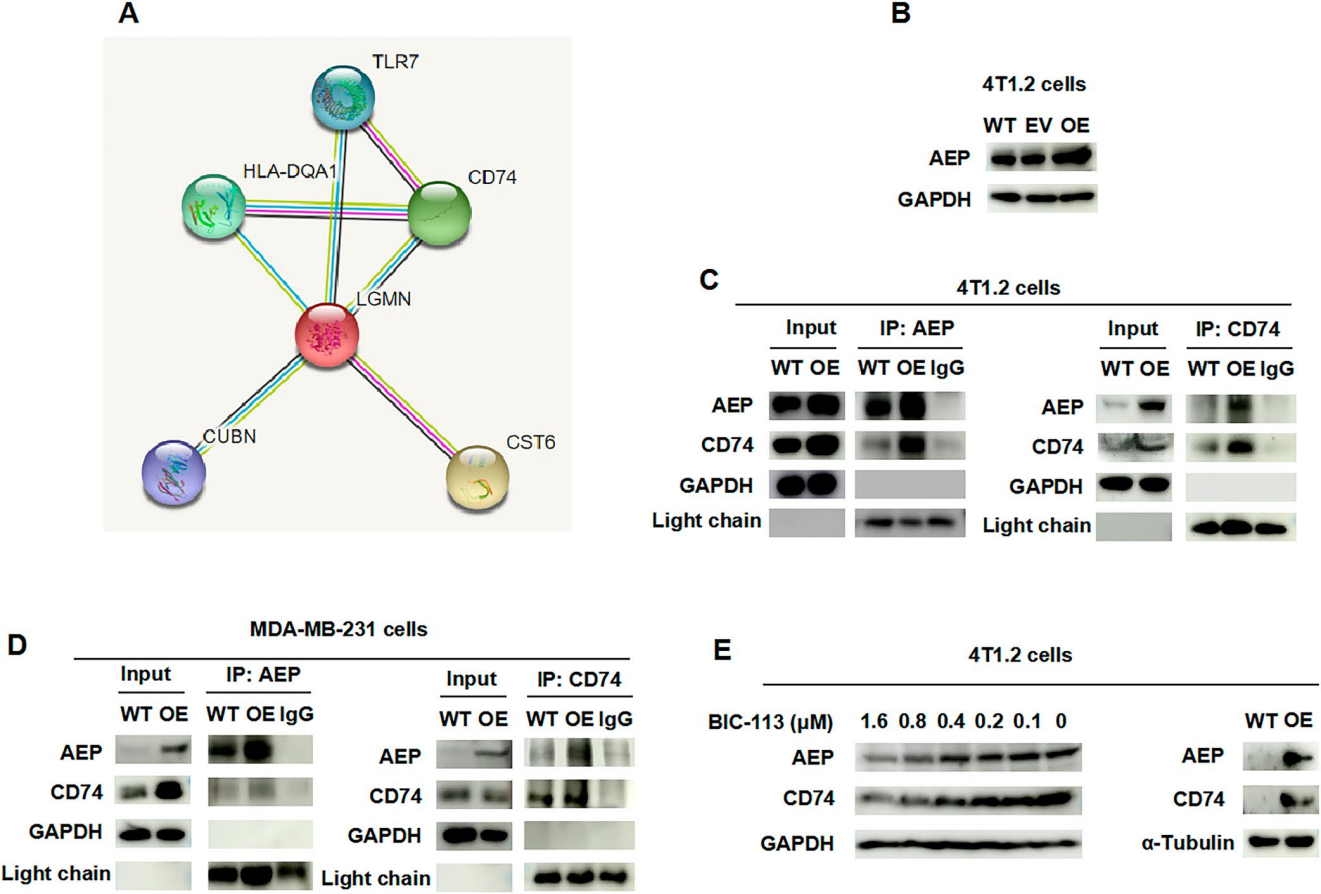
AEP regulates CD74 expression by interacting with CD74. (A) Bioinformatics analysis to find AEP‐interacting proteins; CD74, also known as the invariant chain (Ii), serves as the invariant chain of the MHC class II complex. Structurally, CD74 is a single‐pass type II transmembrane protein composed of an intracellular domain, a transmembrane region, and an extracellular domain. The extracellular domain primarily includes a trimerization region and an MHC class II‐binding region (CLIP region, spanning amino acids 81‐104). AEP recognises and cleaves specific asparagine (Asn) residues within the Ii chain, notably Asn155 located in its luminal domain adjacent to the CLIP region. The cleavage by AEP initiates the stepwise degradation of Ii, thereby regulating the maturation and antigen presentation capacity of MHC class II molecules. Consequently, CD74 is proposed as a putative substrate of AEP. (B) Identification of the 4 T1.2 AEP OE cell line; (C) Interrelationship between 4 T1.2 cell line and CD74 detected by AEP antibody or CD74 antibody; (D) Correlation between MDA‐MB‐231 cell line and CD74 detected by AEP antibody or CD74 antibody; (E) Detection of AEP and CD74 protein expression in 4 T1.2 cells after administration of different concentrations of BIC‐113 or on 4 T1.2 WT and 4 T1.2 AEP OE cells.

To search for proteins that interact with AEP, this study utilised the String database (cn.stringdb.org). The search results indicated a stronger interaction between AEP and CD74. CD74, as an invariant chain of the MHC‐II complex, has been shown to be cleavable by AEP. Subsequently, the protein–protein interaction prediction tool PPA Pred (www.iitm.ac.in) was used to predict the interaction between AEP and the CD74 protein (Figure 1A). The prediction results revealed a predicted binding free energy (Delta G) of −11.32 kcal/mol and a predicted dissociation constant (Kd) of 4.99 × 10^−9^ M. These results suggest that AEP may have a role in tumour metastasis through its interaction with CD74.

To confirm the interaction between AEP and CD74, this study designed and constructed an AEP overexpression (OE) plasmid, which was transfected to obtain a 4 T1.2 AEP overexpression stable transfection cell line (Figure 1B). In 4 T1.2 cells and MDA‐MB‐231 cells, with wild‐type (WT) and OE cell lines selected. The results demonstrated that the amount of protein pulled down by CD74 increased with AEP OE, and the amount of AEP protein pulled down by CD74 antibody also exhibited the same trend as the Input group (Figure 1C,D). Additionally, administering different concentrations of AEP inhibitor BIC‐113 (from low to high dose) led to a decrease in CD74 protein expression with lower AEP protein expression (Figure 1E left). In the AEP OE cell lines, the upregulation of AEP expression also resulted in an increase in CD74 expression (Figure 1E right), indicating that AEP can regulate CD74 protein expression.

### 3.2 AEP Enhances the Migration Ability of Breast Cancer Cells by Regulating CD74

**FIGURE 2:**
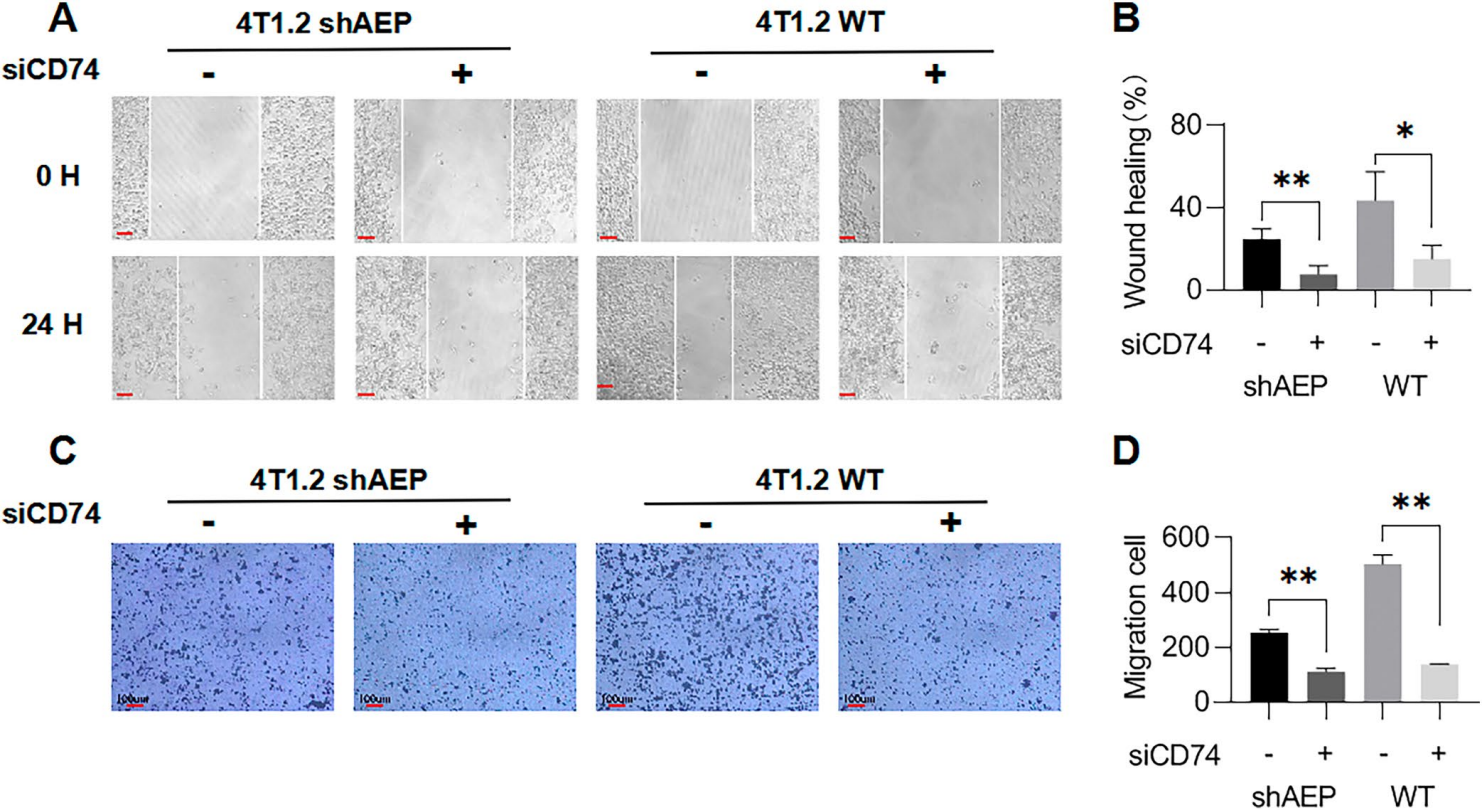
AEP enhances the migration ability of breast cancer cells by regulating CD74. (A) Scratching experiments on different groups of 4 T1.2 cells after administration of siCD74; scale bar is 100 μm; (B) Statistics of the results of scratching experiments on different groups of 4 T1.2 cells; (C) Transwell migration experiments on different groups of 4 T1.2 cells after administration of siCD74; scale bar is 100 μm; (D) Statistics of the results of transwell migration experiments on different groups of 4 T1.2 cells. Compared with the control group, *p < 0.05, **p < 0.01.

WT cell lines displayed a longer migration distance compared to 4 T1.2 shAEP (short hairpin RNA) cells (Figure 2A right). In the same cell lines, the migration ability of cells was significantly inhibited following the administration of siCD74 (small interfering RNA) (*p* < 0.01, Figure 2B). Furthermore, after the administration of siCD74, the migration ability of WT cell lines, which was enhanced by higher levels of AEP compared to shAEP cell lines, was reversed by the knockdown of the CD74 gene (Figure 2A left). Transwell migration experiments verified these findings (Figure 2C,D), indicating that the knockdown of CD74 can reverse the migration ability of breast cancer cells enhanced by AEP, suggesting that AEP can enhance the migration capability of breast cancer cells by regulating CD74.

### 3.3 AEP Enhances Epithelial Mesenchymal Transformation of Breast Cancer Cells by Regulating CD74

**FIGURE 3:**
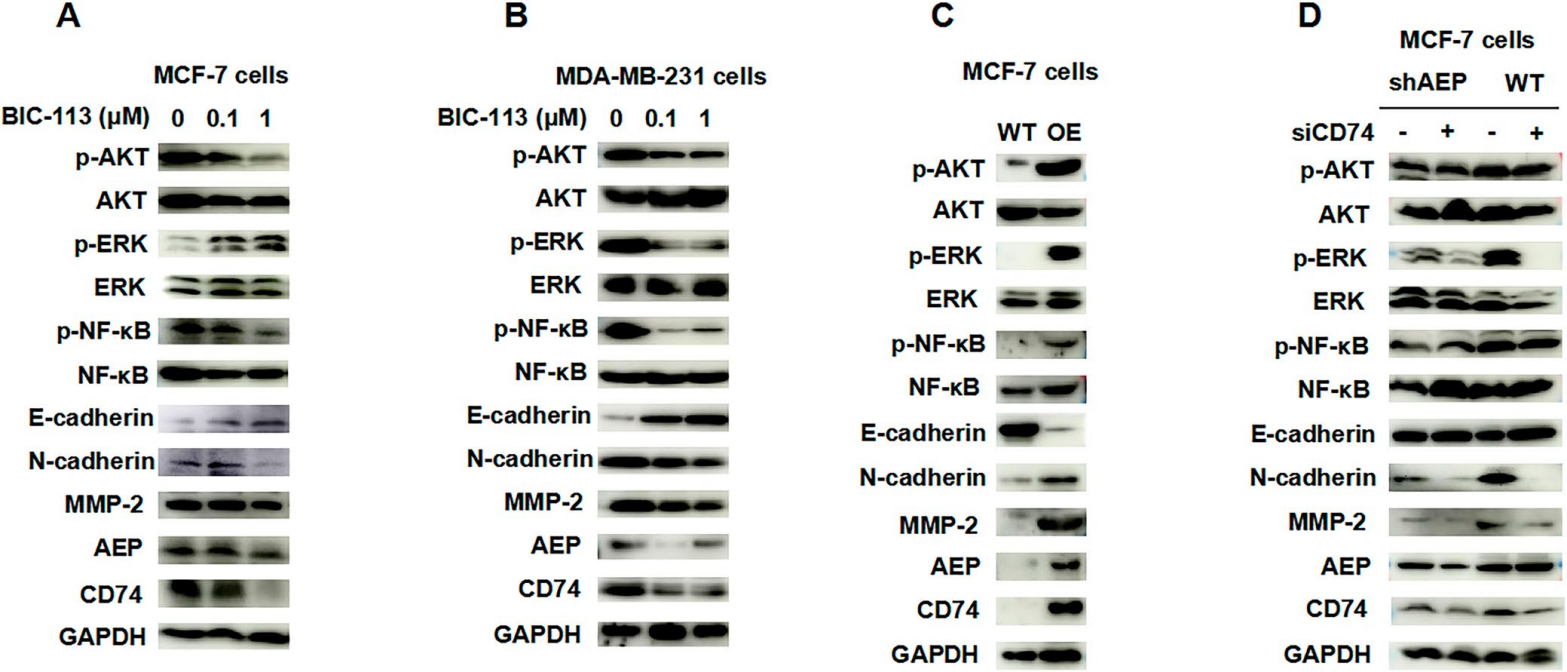
AEP enhances the level of epithelial mesenchymal transition in breast cancer cells by regulating CD74. (A) Expression of related pathway protein after administration of BIC‐113 to MCF‐7 cells; (B) Protein expression of related pathways after administration of BIC‐113 to MDA‐MB‐231 cells; (C) Protein expression of related pathways in MCF‐7 AEP OE cell line and MCF‐7 WT cell line; (D) Protein expression of related pathways after administration of siCD74 to MCF‐7 WT cells and MCF‐7 shAEP cells.

Administer the BIC‐113 in the MCF‐7 cell line and MDA‐MB‐231 cell line, respectively, at 0.1 μM and 1 μM (Figure 3A,B). Compared with the control group, it shows that the expression level of AEP is inhibited by BIC‐113. Corresponding to a decrease in CD74 expression level, the phosphorylation level of AKT and NF‐κB decreases, the expression level of E‐cadherin increases, and the expression of N‐cadherin and MMP‐2 decreases (Figure 3A,B). This indicates that BIC‐113 inhibition of AEP reduces the phosphorylation level of AKT and NF‐κB, as well as the level of EMT. In the AEP overexpressing MCF‐7 cell line, it was observed that upregulation of AEP expression increases the expression of CD74 and activates the phosphorylation of the AKT, ERK, and NF‐κB pathways (Figure 3C). This leads to a decrease in the expression of E‐cadherin and an increase in the expression of N‐cadherin and MMP‐2 (Figure 3C), indicating the promotion of the EMT process.

Subsequently, siCD74 was administered to the MCF‐7 WT and MCF‐7 shAEP cell lines. Compared to the MCF‐7 shAEP cell lines, the phosphorylation levels of the AKT, ERK, and NF‐κB pathways are higher in the MCF‐7 WT cell lines. After the administration of siCD74, the expression levels of p‐ERK were reversed, but the expression levels of p‐AKT and p‐NF‐κB did not show significant changes. Meanwhile, the expression of E‐cadherin was upregulated, while the expression of N‐cadherin and MMP‐2 was reduced (Figure 3D). These results suggest that knocking down CD74 can inhibit the EMT process caused by higher levels of AEP, and this action is achieved through the influence on p‐ERK. Therefore, AEP can activate the phosphorylation of the AKT, ERK, and NF‐κB pathways to promote the EMT process, while CD74 can be regulated by AEP, affecting the EMT process by activating the phosphorylation of the ERK pathway.

### 3.4 Exogenous AEP Enhances the Epithelial‐Mesenchymal Transformation of Breast Cancer Cells

**FIGURE 4:**
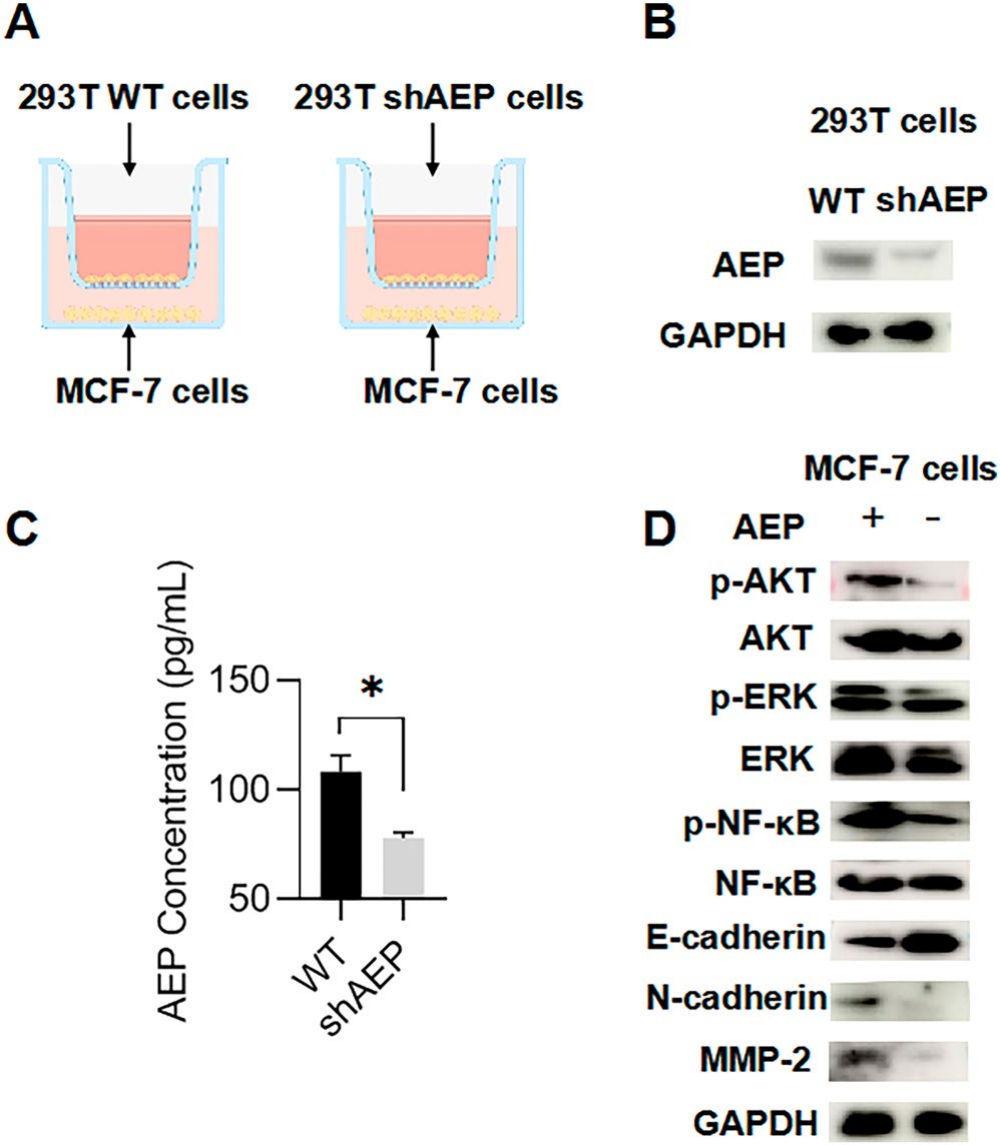
Exogenous AEP enhances the level of epithelial mesenchymal transition in breast cancer cells. (A) Cell co‐culture model; (B) Protein expression of 293 T WT and 293 T shAEP cells; (C) AEP levels in the supernatant of 293 T WT and 293 T shAEP cells; (D) Expression of MCF‐7 cell‐associated proteins after co‐culture with different groups of 293 T cells. Compared with the control group. *p < 0.05.

AEP, as a protease, not only plays a shearing and processing role in tumour cells, enhancing their ability to metastasize and invade, but it is also often excreted into the tumour microenvironment by exosomes of tumour cells to promote metastasis and invasion. To verify whether exogenous AEP affects the EMT level of breast cancer cells, we designed a cell co‐culture model based on the transwell chamber (Figure 4A), using 293 T WT cells and 293 T shAEP cells to co‐culture with MCF‐7 cells. The results show that there is a difference in the expression level of AEP protein between 293 T WT cells and 293 T shAEP cells (Figure 4B), and the supernatant of 293 T WT cells has a higher level of excreted AEP than 293 T shAEP cells by ELISA experiment (Figure 4C). Subsequently, we co‐cultured the two types of cells with MCF‐7 cells separately. The results show that under the higher level of exogenous AEP, the phosphorylation level of AKT, ERK, and NF‐κB pathways, and the EMT level increased in breast cancer cells (Figure 4D), indicating that exogenous AEP can enhance the level of epithelial‐mesenchymal transformation of breast cancer cells and affect the ability of breast cancer cells to metastasize and invade.

### 3.5 Combined Inhibition of AEP and CD74 Can Effectively Reduce the Migration Ability of Breast Cancer Cells and the Level of Epithelial Mesenchymal Transformation

**FIGURE 5:**
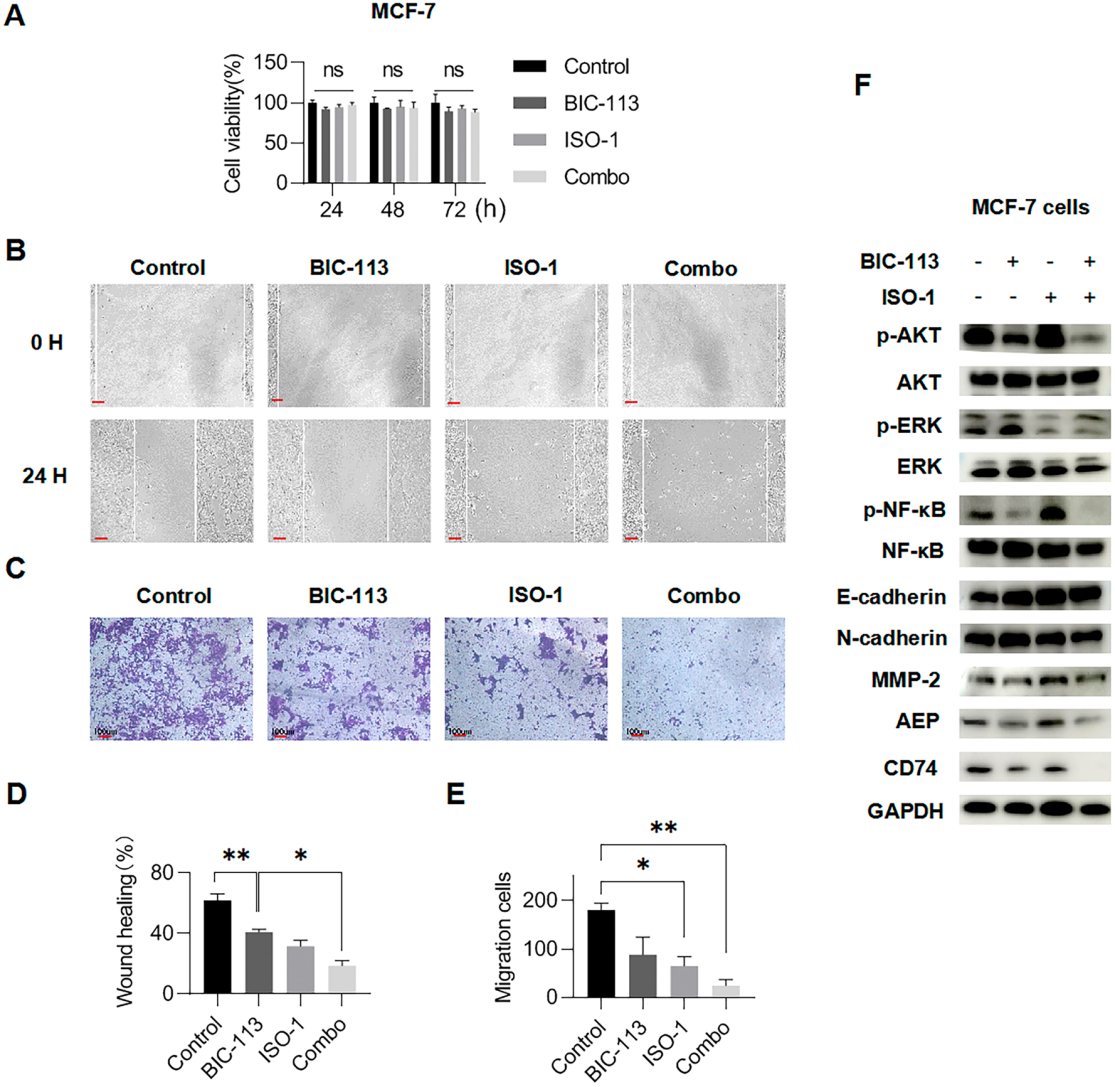
Combined inhibition of AEP and CD74 can reduce the migration ability of breast cancer cells and the level of epithelial mesenchymal transformation. (A) Cell viability of MCF‐7 cell line with different dosing regimens; (B) Results of wound healing assay of MCF‐7 cell line, scale bar is 100 μM; (C) Results of transwell migration assay of MCF‐7 cell line, scale bar is 100 μM; (D, E) Statistics of wound healing assay or transwell migration assay of MCF‐7 cell line; Compared with the control group. *p < 0.05, **p < 0.01, ns *p* > 0.05; (F) Combined inhibition of AEP and CD74 reduces the level of epithelial mesenchymal transition in breast cancer cells.

The inhibitor ISO‐1, a MIF inhibitor, blocks the binding of MIF to CD74 and inhibits CD74 signal transduction [20]. We used 10 μM ISO‐1 as a single drug group and 1 μM BIC‐113 as another single drug group. In addition, we employed 10 μM ISO‐1 and 1 μM BIC‐113 as a combo group. The results demonstrated that the three drug groups showed no significant effect on the proliferation ability of MCF‐7 cells (Figure 5A). Subsequently, we conducted a transwell migration experiment on the MCF‐7 cell line using the aforementioned drug groups (Figure 5B,C). The results revealed that both the BIC‐113 and ISO‐1 single drug groups inhibited the migration of MCF‐7 cells (*p* < 0.05), while the combo group exhibited a more significant inhibition of MCF‐7 cell migration (*p* < 0.01) (Figure 5B–E). These findings suggest that the combined inhibition of AEP and CD74 can reduce the migration ability of breast cancer cells more effectively than inhibiting either AEP or CD74 alone. After treatment with BIC‐113 or ISO‐1, breast cancer cells showed a decrease in the expression of EMT‐related proteins (Figure 5F). However, the reduction in EMT levels was more significant in the combo group compared to the two individual drugs. It is important to note that BIC‐113 primarily inhibited the expression of p‐AKT and p‐NF‐κB, but had no significant inhibitory effect on p‐ERK. On the other hand, ISO‐1 mainly inhibited the expression of p‐ERK, not p‐AKT and p‐NF‐κB, which aligns with the results of CD74 siRNA on the mentioned pathway. In the combo group, the expression of all three phosphorylated proteins was inhibited, indicating that the combination of BIC‐113 and ISO‐1 can effectively lower the EMT level of breast cancer cells compared to each drug alone.

### 3.6 Combined Inhibition of AEP and CD74 for Overcoming Lung Metastasis in Breast Cancer

**FIGURE 6:**
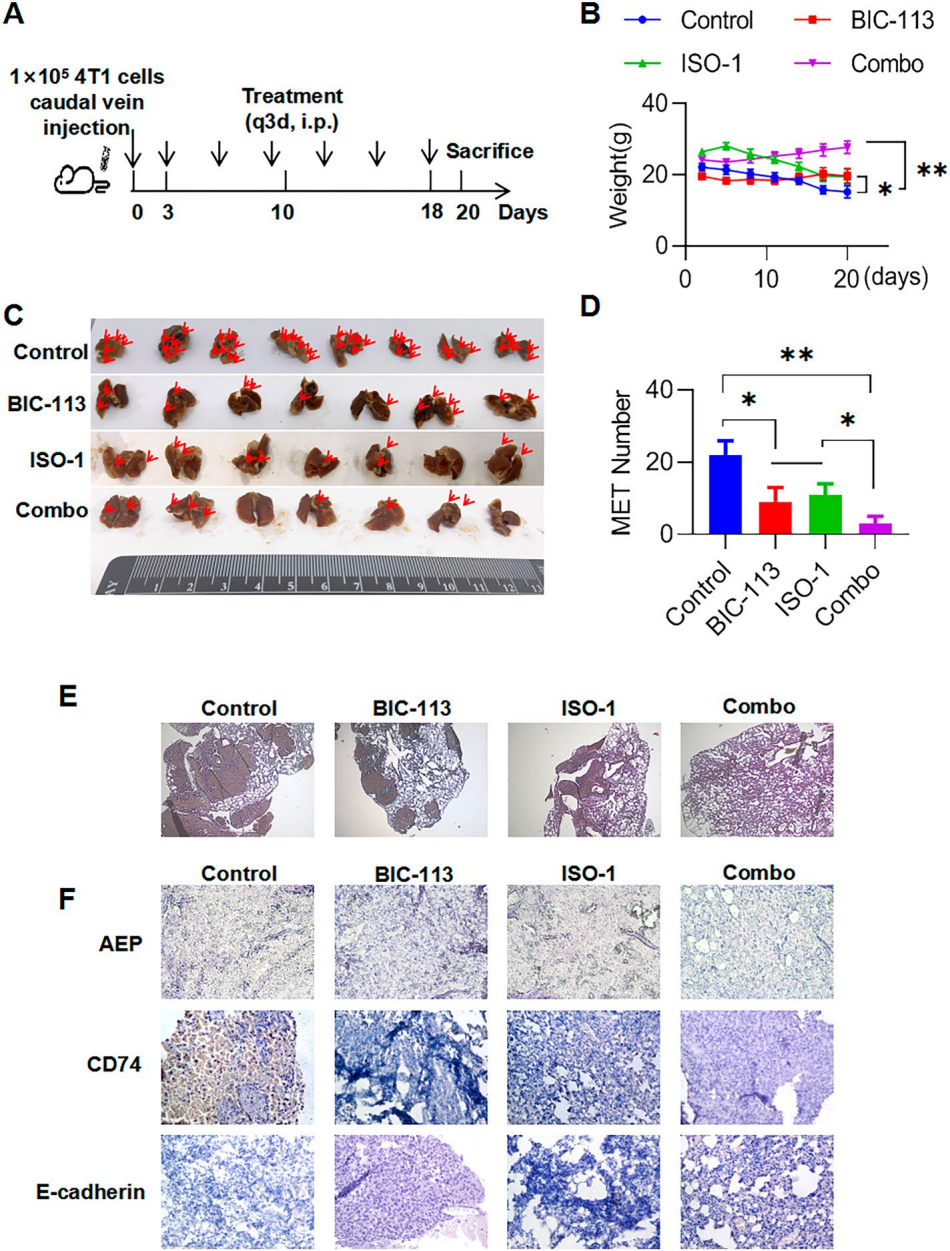
Combined inhibition of AEP and CD74 to overcome breast cancer lung metastasis. (A) Experimental protocol for breast cancer lung metastasis animals; (B) Body weight statistics of mice in each group; (C) Photographs of lung metastases in each group of mice, with red arrows indicating lung metastases; (D) The number of lung metastases in each group of mice; (E) HE staining of lung tissues in each group of mice; (F), Immunohistochemistry of AEP, CD74 and E‐cadherin in lung tissues of mice in each group. Compared with the control group: *p < 0.05, **p < 0.01.

The results showed that when BIC‐113 or ISO‐1 was given alone, the weight of mice in the control group significantly improved (*p* < 0.05) (Figure 6B). Meanwhile, the weight of mice in the Combo group improved significantly (*p* < 0.01). This indicates that compared to BIC‐113 or ISO‐1 treatment alone, the combined inhibition of AEP and CD74 could better improve the prognosis of mice with lung metastasis from breast cancer and had no significant toxic effect on their weight. The mice were killed on the 20th day, and the whole lungs of mice in each group were taken to observe and record the lung metastasis of breast cancer (Figure 6A). It was observed that the number of lung metastases in the BIC‐113 or ISO‐1 single drug group significantly improved compared to the control group (*p* < 0.05), while the number of lung metastases in the Combo group significantly reduced (*p* < 0.01) (Figure 6C,D). This suggests that the combined inhibition of AEP and CD74 can effectively overcome lung metastasis of breast cancer and improve the prognosis of mice. HE staining was used to observe breast cancer metastasis in lung tissue. The control group mice showed obvious solid breast cancer metastasis in the lung tissue, while the BIC‐113 or ISO‐1 group showed relatively reduced metastasis. The Combo group showed no obvious lung metastasis in the lung tissue (Figure 6E). After BIC‐113 administration, the expression of AEP in the metastatic focus of breast cancer in the lung tissue of mice was weaker than that in the control group. The expression of AEP in the ISO‐1 group was not reduced, and the expression of AEP in the Combo group was also less (Figure 6F upper line). In the immunohistochemistry of CD74, both the Combo group and BIC‐113 group showed decreased expression compared to the control group (Figure 6F middle line). Similarly, in the immunohistochemistry of E‐cadherin, all three treatment strategies increased the expression compared to the control group, with the Combo group having the highest level of E‐cadherin expression (Figure 6F lower line). These results indicate that the combined inhibition of AEP and CD74 in animals can effectively overcome lung metastasis of breast cancer and improve the prognosis of mice more than CD74 alone.

### 3.7 Combined Inhibition of AEP and CD74 Showed No Significant Visceral Toxicity

**FIGURE 7:**
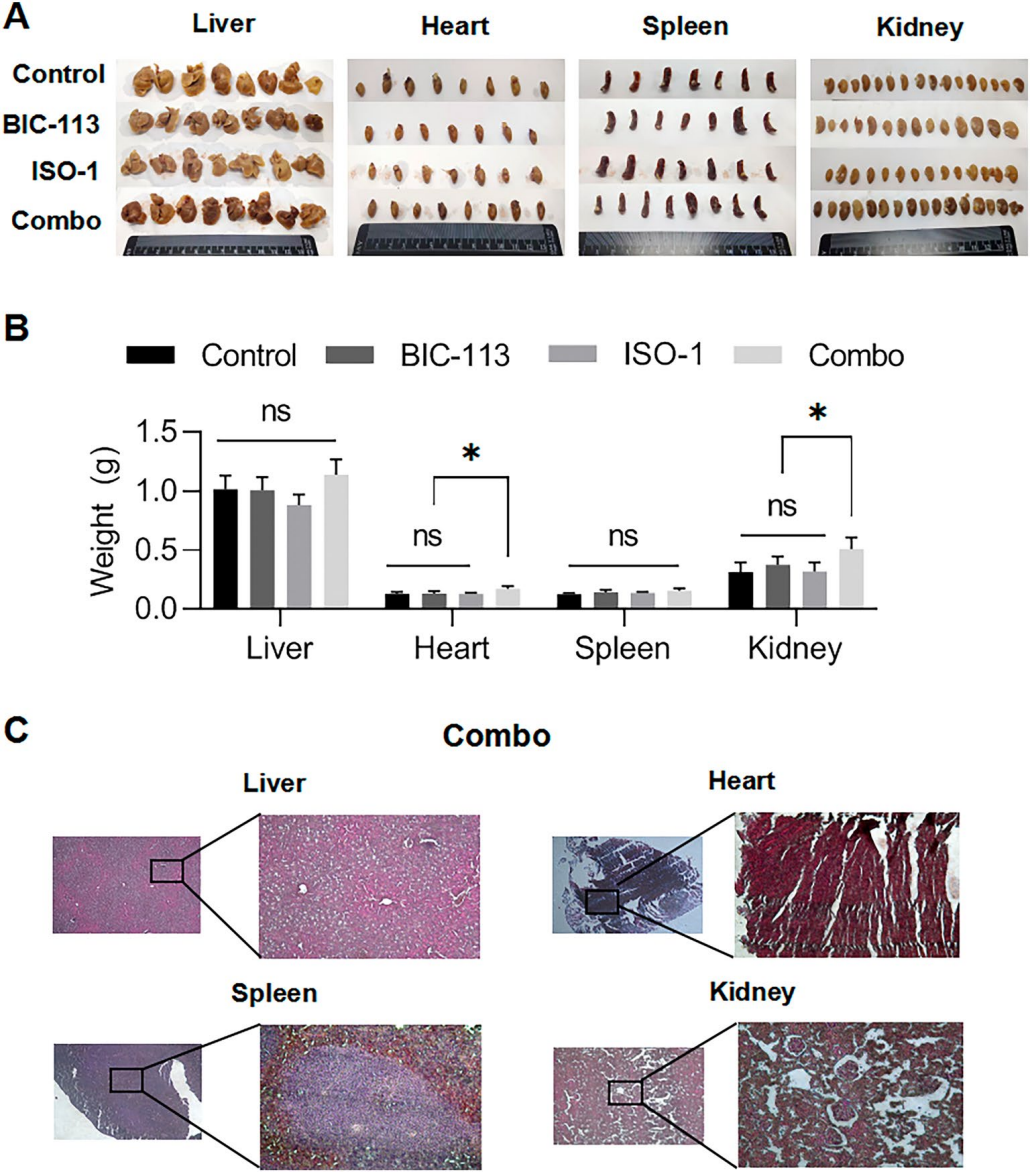
Combined inhibition of AEP and CD74 without significant visceral toxicity. (A) Photographs of liver, heart, spleen, and kidney of mice in each group; (B) Weight statistics of liver, heart, spleen, and kidney of mice in each group; (C) HE staining of liver, heart, spleen, and kidney in Combo group. Compared with the control group. *p < 0.05, ns *p* > 0.05.

This study also conducted sampling analysis on the liver, heart, spleen, and kidney of each group of mice (Figure 7A). The results show that in this model of lung metastasis of breast cancer, there is no obvious liver metastasis in each group of mice. After drug treatment, the liver weight of the mice in each group did not change (Figure 7B liver). HE staining of the liver tissue in the Combo group showed no significant damage (Figure 7C liver). Regarding the heart, the control group, BIC‐113 monotherapy group, and ISO‐1 monotherapy group showed a significant reduction in heart size compared to the Combo group (Figure 7B heart). HE staining of the Combo group showed no significant damage to myocardial cells (Figure 7C heart). As for the spleen, the three treatment regimens did not cause splenomegaly (Figure 7B spleen), and HE staining in the Combo group showed no significant damage to the spleen tissue, indicating that none of the three treatment regimens caused severe inflammation (Figure 7C spleen). In terms of the kidneys, the control group, BIC‐113 monotherapy group, and ISO‐1 monotherapy group had significantly reduced kidney size compared to the Combo group (Figure 7B kidney). However, HE staining of the Combo group showed no significant atrophy or enlargement of the glomeruli, and their morphology appeared normal (Figure 7C kidney). The difference in heart and kidney weight between the other three groups and the Combo group may be related to tumour‐induced weight loss in mice. These results indicate that the combined inhibition of AEP and CD74 does not cause significant visceral toxicity and is safe.

## 4 Discussion

The challenge of breast cancer treatment lies in resisting in vivo metastasis and enhancing the effectiveness of anti‐tumour treatment. Currently, the selection of targeted drugs for metastasis is mainly based on different types of breast cancer, and there has been progress in innovative therapies such as immunotherapy for resisting breast cancer metastasis [21]. Therefore, there is great hope for developing innovative therapies for breast cancer metastasis. AEP, as a member of the C13 family of lysosomal cysteine proteases, plays a role in protein splicing and processing in organisms. It is highly expressed during tumour development and has involvement in tumour‐related physiological and biochemical reactions [22]. The development of AEP‐targeted therapeutics has been actively pursued due to its multifaceted roles in oncogenesis and disease progression. Despite compelling preclinical evidence of AEP's involvement in tumorigenic pathways, no AEP‐specific inhibitors have progressed to clinical trials. This translational gap may stem from the incomplete suppression of metastasis and invasive phenotypes through AEP monotherapy, underscoring the limitations of single‐target strategies [9]. To address this, future investigations should prioritise combinatorial approaches integrating AEP inhibitors with complementary agents, or exploit AEP's tumour‐selective overexpression for precision medicine applications. Notably, innovative drug design paradigms, such as AEP‐directed antibody‐drug conjugates (ADCs) or protease‐activated delivery systems, have shown preclinical promise. For example, paclitaxel‐based AEP‐targeting drug conjugates exhibit potent anti‐proliferative effects in colorectal (HCT116) and breast cancer (MCF‐7) models, with demonstrated efficacy in suppressing pulmonary metastasis and tumour recurrence in vivo [23]. In parallel, CD74 has emerged as a dual‐modality target influencing both tumour biology and immune regulation. Current therapeutic strategies encompass: (1) MIF/CD74 small‐molecule inhibitors, (2) monoclonal antibodies against MIF/CD74 axis components, (3) siRNA‐mediated silencing, and (4) functional disruptors of CD74 signalling. While CD74‐specific inhibitors remain elusive, multiple MIF‐targeted agents have entered clinical evaluation. The isoquinoline derivative ISO‐1 exemplifies this category, demonstrating dual anti‐neoplastic and immunomodulatory properties through competitive MIF binding, effectively suppressing CD74‐mediated signalling, reducing pro‐inflammatory cytokine secretion (TNF, IFN‐γ, IL‐4, IL‐17), and inhibiting glioma proliferation [24]. These mechanistic insights provide a rationale for exploring synergistic combinations of AEP inhibitors with MIF/CD74 pathway modulators, a strategy that may enhance therapeutic efficacy while mitigating compensatory resistance mechanisms.

**FIGURE 8:**
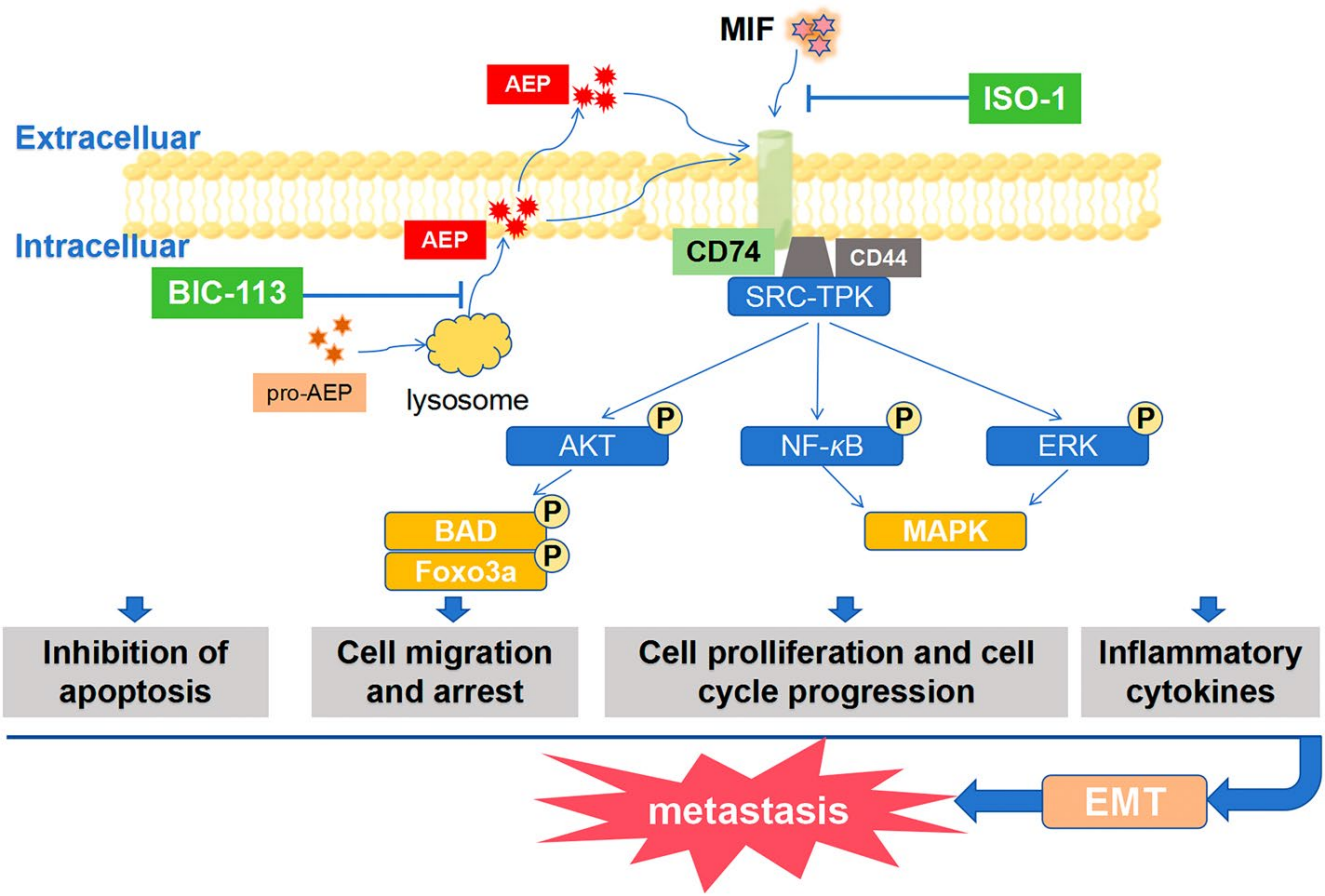
Schematic diagram of mechanism of action and pathway illustrations.

CD74, also known as the invariant chain (Ii), serves as the invariant chain of the MHC class II complex and functions as a receptor for the macrophage migration inhibitory factor (MIF). First identified in the 1970s through co‐immunoprecipitation with the MHC class II complex, CD74 is an evolutionarily highly conserved type II transmembrane glycoprotein [25]. AEP initiates the stepwise degradation of Ii by recognising and cleaving critical asparagine (Asn) residues within the Ii chain. AEP predominantly cleaves at Asn76 and Asn155, with Asn155 located in the luminal domain of Ii adjacent to the CLIP region (amino acids 81–104). This proteolytic activity by AEP represents the initial step in Ii chain degradation, enabling subsequent proteases (e.g., cathepsins S/L) to further process the CLIP region. By cleaving the Ii chain, AEP facilitates the liberation of the antigen‐binding groove of MHC class II molecules, thereby allowing their engagement with exogenous antigenic peptides and regulating the maturation and antigen presentation capacity of MHC class II molecules. This regulatory mechanism is particularly critical in professional antigen‐presenting cells (APCs), including B lymphocytes and dendritic cells [26]. This study investigated the impact of AEP on the metastasis of breast cancer cells. Protein interaction analysis revealed a potential relationship between AEP and CD74 in influencing the EMT process of breast cancer cells. Immunoprecipitation confirmed the association between AEP and CD74, further demonstrating that AEP activates the phosphorylation of the ERK pathway through CD74. This activation improves the migration ability and EMT level of breast cancer cells. Additionally, exogenous AEP was found to enhance the EMT level of breast cancer cells. CD74, known as an invariant chain of the MHC‐II complex and a receptor for MIF, is frequently associated with high expression and poor prognosis during tumour development [27]. CD74 primarily promotes tumour progression through its signalling function, making it a potential tumour marker or targeted drug target [28] Due to the absence of targeted inhibitors for CD74, the MIF inhibitor ISO‐1 was chosen to inhibit CD74 function by occupying the binding domain of MIF. This inhibition prevents the binding between MIF and CD74, effectively inhibiting CD74 function. Based on the discovery that AEP influences the metastatic and invasive ability of breast cancer cells through CD74 and previous findings that AEP alone does not completely inhibit bone metastasis, this study proposes the hypothesis that the combined inhibition of AEP and CD74 can significantly inhibit breast cancer metastasis (Figure 8). Subsequent in vitro experiments, including transwell migration and Western Blot experiments, demonstrated that the combined inhibition of AEP and CD74 reduces the migration ability and EMT level of breast cancer cells. In a breast cancer lung metastasis model, it was observed that the combined scheme successfully resists lung metastasis, improves the prognosis of mice, and does not display obvious toxicity. Breast cancer metastasis mechanisms have witnessed significant therapeutic advancements, with emerging agents demonstrating targeted efficacy across distinct molecular pathways. CXCR4 antagonists (e.g., plerixafor) modulate tumour microenvironment (TME) dynamics by disrupting the CXCR4/CXCL12 chemotactic axis, thereby impeding metastatic niche colonisation through interference with tumour cell tropism. TGF‐β signalling inhibitors such as galunisertib exert dual anti‐metastatic effects by suppressing both epithelial‐mesenchymal plasticity (EMP) and pre‐metastatic niche maturation. Immune checkpoint blockade strategies, exemplified by pembrolizumab in PD‐L1+ triple‐negative breast carcinoma (TNBC), have redefined therapeutic paradigms through robust inhibition of distant metastatic dissemination. Notably, AEP‐targeted therapeutics and CD74/MIF axis inhibitors present mechanistically distinct intervention points. Preclinical investigations have demonstrated AEP inhibition confers specific protection against osteolytic metastasis initiation and progression, a finding of particular clinical relevance given the propensity of breast malignancies for skeletal colonisation. This unique mechanistic profile positions these agents as viable second‐line alternatives for patients exhibiting primary resistance to conventional targeted therapies. Therefore, combination therapy utilising AEP‐targeted inhibitors and CD74/MIF pathway inhibitors may provide comprehensive inhibition of distant organ metastasis in breast cancer, potentially emerging as a promising clinical strategy for future therapeutic development.

To sum up, this study found that inhibiting AEP can resist bone metastasis of breast cancer. Furthermore, AEP was found to enhance the metastatic and invasive ability of breast cancer cells through CD74 regulation. Lastly, the combined inhibition of AEP and CD74 was proven to safely and effectively combat lung metastasis of breast cancer, providing novel insights for clinical treatment. This study clarifies the molecular mechanism of AEP promoting breast cancer metastasis by regulating CD74, explores the therapeutic effect and safety of AEP and CD74 combined inhibition on breast cancer metastasis, and provides new targets and ideas for breast cancer metastasis treatment.

## Author Contributions

**Junsong Chen:** investigation (equal), methodology (equal), writing – original draft (equal). **Wenke Xu:** formal analysis (equal), investigation (equal). **Luyang Meng:** data curation (equal). **Xin Zhang:** data curation (equal), software (equal). **Mengyao Lin:** writing – original draft (equal). **Sheng Zhang:** writing – original draft (equal). **Yi Liu:** funding acquisition (equal), resources (equal). **Fang Guo:** resources (equal), supervision (equal), writing – review and editing (equal).

## Ethics Statement

This study was approved by Shanghai Jiao Tong University Animal Ethics Committee (approval number: 202201078).

## Consent

The authors have nothing to report.

## Conflicts of Interest

The authors declare no conflicts of interest.

## Data Availability

The data that support the findings of this study are available from the corresponding author upon reasonable request.

## References

[1] Y. L. Li , Y. C. Qin , L. Y. Tang , et al., “Patient and Care Delays of Breast Cancer in China,” Cancer Research and Treatment 51, no. 3 (2019): 1098–1106, 10.4143/crt.2018.386. 30428639 PMC6639234

[2] G. J. R. Cook , “Imaging of Bone Metastases in Breast Cancer,” Seminars in Nuclear Medicine 52, no. 5 (2022): 531–541, 10.1053/j.semnuclmed.2022.01.005. 35236615 PMC7616189

[3] J. Lengrand , I. Pastushenko , S. Vanuytven , et al., “Pharmacological Targeting of Netrin‐1 Inhibits EMT in Cancer,” Nature 620, no. 7973 (2023): 402–408, 10.1038/s41586-023-06372-2. 37532929 PMC7615210

[4] R. Gupta , R. Ponangi , and K. G. Indresh , “Role of Glycosylation in Breast Cancer Progression and Metastasis: Implications for miRNA, EMT and Multidrug Resistance,” Glycobiology 33, no. 7 (2023): 545–555, 10.1093/glycob/cwad046. 37283470

[5] E. Nolan , G. J. Lindeman , and J. E. Visvader , “Deciphering Breast Cancer: From Biology to the Clinic,” Cell 186, no. 8 (2023): 1708–1728, 10.1016/j.cell.2023.01.040. 36931265

[6] B. D. Reddy , N. M. Beeraka , C. M. K. Chitturi , and S. V. Madhunapantula , “An Overview of Targeting Legumain for Inhibiting Cancers,” Current Pharmaceutical Design 27, no. 31 (2021): 3337–3348, 10.2174/1381612826666201125111625. 33238867

[7] Y. Lin , Y. Qiu , C. Xu , et al., “Functional Role of Asparaginyl Endopeptidase Ubiquitination by TRAF6 in Tumor Invasion and Metastasis,” Journal of the National Cancer Institute 106, no. 4 (2014): dju012, 10.1093/jnci/dju012. 24610907

[8] W. Zhang and Y. Lin , “The Mechanism of Asparagine Endopeptidase in the Progression of Malignant Tumors: A Review,” Cells 10, no. 5 (2021): 1153, 10.3390/cells10051153. 34068767 PMC8151911

[9] K. Lei , S. S. Kang , E. H. Ahn , et al., “C/EBPβ/AEP Signaling Regulates the Oxidative Stress in Malignant Cancers, Stimulating the Metastasis,” Molecular Cancer Therapeutics 20, no. 9 (2021): 1640–1652, 10.1158/1535-7163.MCT-21-0019. 34158346

[10] X. Xu , M. Liu , K. Peng , Y. Yu , and T. Liu , “Asparaginyl Endopeptidase Contributes to Cetuximab Resistance via MEK/ERK Signaling in RAS Wide‐Type Metastatic Colorectal Cancer,” Clinical & Translational Oncology 25, no. 3 (2023): 776–785, 10.1007/s12094-022-02986-6. 36609651 PMC9941237

[11] J. Chen , W. Xu , K. Song , et al., “Legumain Inhibitor Prevents Breast Cancer Bone Metastasis by Attenuating Osteoclast Differentiation and Function,” Bone 169 (2023): 116680, 10.1016/j.bone.2023.116680. 36702335

[12] Q. Qi , O. Obianyo , Y. Du , H. Fu , S. Li , and K. Ye , “Blockade of Asparagine Endopeptidase Inhibits Cancer Metastasis,” Journal of Medicinal Chemistry 60, no. 17 (2017): 7244–7255, 10.1021/acs.jmedchem.7b00228. 28820254 PMC5871875

[13] J. Xiong , Z. Zhang , and K. Ye , “C/EBPβ/AEP Signaling Drives Alzheimer's Disease Pathogenesis,” Neuroscience Bulletin 39, no. 7 (2023): 1173–1185, 10.1007/s12264-023-01025-w. 36735152 PMC10313643

[14] M. T. Bozza , L. Lintomen , J. Z. Kitoko , C. N. Paiva , and P. C. Olsen , “The Role of MIF on Eosinophil Biology and Eosinophilic Inflammation,” Clinical Reviews in Allergy and Immunology 58, no. 1 (2020): 15–24, 10.1007/s12016-019-08726-z. 30680604

[15] J. Wang , J. Hong , F. Yang , et al., “A Deficient MIF‐CD74 Signaling Pathway May Play an Important Role in Immunotherapy‐Induced Hyper‐Progressive Disease,” Cell Biology and Toxicology 39, no. 3 (2023): 1169–1180, 10.1007/s10565-021-09672-3. 34797429

[16] L. Cao , X. Wang , X. Liu , et al., “Tumor Necrosis Factor α‐Dependent Lung Inflammation Promotes the Progression of Lung Adenocarcinoma Originating From Alveolar Type II Cells by Upregulating MIF‐CD74,” Laboratory Investigation 103, no. 3 (2023): 100034, 10.1016/j.labinv. 36925198

[17] L. Yan , M. Wu , T. Wang , et al., “Breast Cancer Stem Cells Secrete MIF to Mediate Tumor Metabolic Reprogramming That Drives Immune Evasion,” Cancer Research 84, no. 8 (2024): 1270–1285, 10.1158/0008-5472.CAN-23-2390. 38335272

[18] L. Klemke , T. De Oliveira , D. Witt , et al., “Hsp90‐Stabilized MIF Supports Tumor Progression via Macrophage Recruitment and Angiogenesis in Colorectal Cancer,” Cell Death & Disease 12, no. 2 (2021): 155, 10.1038/s41419-021-03426-z. 33542244 PMC7862487

[19] I. Cotzomi‐Ortega , O. Nieto‐Yañez , I. Juárez‐Avelar , et al., “Autophagy Inhibition in Breast Cancer Cells Induces ROS‐Mediated MIF Expression and M1 Macrophage Polarization,” Cellular Signalling 86 (2021): 110075, 10.1016/j.cellsig.2021.110075. 34229086

[20] X. Shi , Y. Wu , H. Ni , M. Li , B. Qi , and Y. Xu , “Macrophage Migration Inhibitory Factor (MIF) Inhibitor iSO‐1 Promotes Staphylococcal Protein A‐Induced Osteogenic Differentiation by Inhibiting NF‐κB Signaling Pathway,” International Immunopharmacology 115 (2023): 109600, 10.1016/j.intimp. 36577150

[21] T. G. Lyons , “Targeted Therapies for Triple‐Negative Breast Cancer,” Current Treatment Options in Oncology 20, no. 11 (2019): 82, 10.1007/s11864-019-0682-x. 31754897

[22] T. Zhao , Y. Liu , Y. Hao , et al., “Esomeprazole Inhibits the Lysosomal Cysteine Protease Legumain to Prevent Cancer Metastasis,” Investigational New Drugs 39, no. 2 (2021): 337–347, 10.1007/s10637-020-01011-3. 32978718

[23] S. Balamkundu and C. F. Liu , “Lysosomal‐Cleavable Peptide Linkers in Antibody‐Drug Conjugates,” Biomedicine 11, no. 11 (2023): 3080, 10.3390/biomedicines11113080. PMC10669454 38002080

[24] R. Li , D. Li , H. Wang , et al., “Exosomes From Adipose‐Derived Stem Cells Regulate M1/M2 Macrophage Phenotypic Polarization to Promote Bone Healing via miR‐451a/MIF,” Stem Cell Research & Therapy 13, no. 1 (2022): 149, 10.1186/s13287-022-02823-1. 35395782 PMC8994256

[25] K. Sumaiya , D. Langford , K. Natarajaseenivasan , and S. Shanmughapriya , “Macrophage Migration Inhibitory Factor (MIF): A Multifaceted Cytokine Regulated by Genetic and Physiological Strategies,” Pharmacology & Therapeutics 233 (2022): 108024, 10.1016/j.pharmthera.2021.108024. 34673115

[26] B. Manoury , D. Mazzeo , D. N. Li , et al., “Asparagine Endopeptidase Can Initiate the Removal of the MHC Class II Invariant Chain Chaperone,” Immunity 18, no. 4 (2003): 489–498, 10.1016/s1074-7613(03)00085-2. 12705852

[27] D. Loreth , M. Schuette , J. Zinke , et al., “CD74 and CD44 Expression on CTCs in Cancer Patients With Brain Metastasis,” International Journal of Molecular Sciences 22, no. 13 (2021): 6993, 10.3390/ijms22136993. 34209696 PMC8268634

[28] X. Xu , Y. Li , R. Xu , et al., “CD74‐ROS1 L2026M Mutant Enhances Autophagy Through the MEK/ERK Pathway to Promote Invasion, Metastasis and Crizotinib Resistance in Non‐Small Cell Lung Cancer Cells,” FEBS Journal 291, no. 6 (2024): 1199–1219, 10.1111/febs.17032. 38148635

